# Is the effectiveness of patellofemoral bracing modified by patellofemoral alignment and trochlear morphology?

**DOI:** 10.1186/s12891-017-1524-2

**Published:** 2017-04-21

**Authors:** Xi Zhang, Jillian P. Eyles, Joanna Makovey, Matthew J. Williams, David J. Hunter

**Affiliations:** 1 University of Sydney, Sydney, Australia; 2 Department of Rheumatology, Royal North Shore Hospital and Northern Clinical School, University of Sydney, Pacific Highway, St Leonards, NSW 2065 Australia; 3 Kolling Institute of Medical Research, Institute of Bone and Joint Research, University of Sydney, Sydney, Australia; 4 Physiotherapy Department, Royal North Shore Hospital, Sydney, Australia

**Keywords:** Patellofemoral osteoarthritis, Patella alignment, Patellofemoral bracing

## Abstract

**Background:**

This study was performed to determine if the effectiveness of patellofemoral bracing as a treatment for patellofemoral osteoarthritis is influenced by patellofemoral joint alignment and trochlear morphology. We hypothesized that those with more extreme patellar malalignment would benefit more from bracing.

**Methods:**

Thirty-eight patients who had received bracing as part of a comprehensive treatment plan for patellofemoral osteoarthritis were selected for this study. Ten measures of patellar alignment were taken from X-rays. These alignment measures were divided into percentile groups (tertiles) for contingency table analysis. Treatment outcome was measured by Western Ontario and Macmasters Universities Osteoarthritis Index (WOMAC) scores and these were dichotomised into two groups according to “Improved” or “Not Improved” according to the minimum clinically important difference (MCID). Spearman’s rho test was performed for continuous variables and Fisher’s exact test was performed for correlation between tertile groups and MCID categories.

**Results:**

Thirty-eight patients (9 male and 29 female) between the ages of 51 to 89 were included in this study. WOMAC scores ranged from −25 to 41.67, with a mean change of −3.97, 31.6, 44.7 and 31.6% of patients falling into the “Improved” group for Global, Pain and Function scores respectively. We found a non-significant trend shown (*p =* 0.058, correlation coefficient 0.31) between bisect offset and change in WOMAC global, indicating a trend for higher change in WOMAC scores with increasing bisect offset. Statistically significant correlations were found between mean MCID categories for the WOMAC global and function groups when analysed against percentile groups for bisect offset (*p <* 0.01) and patellar subluxation distance (*p <* 0.05), indicating those in higher percentile groups were more likely not to improve after six months.

**Conclusion:**

Higher bisect offset and patellar subluxation distance measures were associated with poorer outcomes. However, due to the limited sample size, more studies are required to fully examine this relationship.

## Background

Osteoarthritis (OA) is the most common form of arthritis [1], with the knee being affected frequently. Knee OA is a major contributor to pain and disability among the elderly [1]. The majority of research has focused on the tibiofemoral compartment, however patellofemoral (PF) osteoarthritis coexists with tibiofemoral OA in up to 65% of patients [2], and there is a growing body of evidence that suggests patellofemoral osteoarthritis (PF OA) is strongly linked to pain and disability [3]. Patellar alignment has been reported to be correlated with symptom severity and is a radiographic predictor for progression of PF OA [3, 4]. Although easily measured radiographically, the delineation between normal variation and pathological patellar malalignment is not well understood [5], and may have significant implications for personalising treatments such as knee bracing.

Bracing is a popular treatment for chronic knee pain, being widely accessible and relatively inexpensive [6]. Tibiofemoral OA has been the focus of most studies to date. Some studies have demonstrated significant improvement in symptoms, but the evidence for bracing in PF OA is less convincing [6–8]. Previous research has shown medially-directed patellar taping to have beneficial outcomes associated with chronic knee pain [6, 8], and is postulated to derive its effects by increasing joint surface contact area and reducing stress on vulnerable structures [6]. Patellar bracing provides certain advantages over taping, including longer equipment life due to reusable materials, and lack of possible allergic dermatitis to adhesives. As bracing provides similar biomechanical effects as taping, such as increasing joint contact area [9], it should similarly reduce symptoms, but the results thus far have been more conclusive for the use of bracing in patellar instability, and less convincing for PF OA [6, 10].

Considering present evidence that variations in patellar alignment are both widespread and a significant contributor towards PF OA progression and symptoms, it would be highly beneficial to determine if the extent of malalignment influences the effect of bracing efficacy. A randomised control trial by Callaghan et al. found PF bracing had a moderate effect on knee pain, but did not account for patellar alignment [11]. Thus far, there have been no studies examining the relationship between degree of malalignment and its effects on treatment outcomes. A correlation between patellar alignment and treatment outcome may help guide future individualised treatment options based on radiographic measures.

This study was designed to examine if patellofemoral alignment parameters modified the efficacy of bracing as a treatment option for PF OA, as part of a comprehensive treatment approach. It examined the relationships between patellofemoral alignment parameters and whether study participants were “improved” or “not improved”. We hypothesized that those with more extreme patellar malalignment would benefit from bracing more than those with less extreme malalignment.

## Methods

### Study sample

This is an ancillary analysis from patients who attended the Osteoarthritis Chronic Care Program (OACCP) at Royal North Shore Hospital, Sydney. The OACCP is a multi-centre, multidisciplinary program, specialising in non-surgical management of knee and hip osteoarthritis. Program participants were assessed by a musculoskeletal (MSK) coordinator who was a physiotherapist for progressive resistance and aerobic-based exercise prescription at baseline, 12, 26 and 52 weeks. All participants were reviewed by a rheumatologist for management of pain medications and supplement advice. The MSK coordinator made further referrals to the following health practitioners according to clinical need; dietician for weight management, occupational therapist for optimisation of functional tasks and provision of assistive devices, a social worker to address psychosocial needs and an orthotist if foot orthoses and/or appropriate knee bracing were required. All patients who were braced also received exercise and weight management interventions from allied health, and pain medication review by rheumatologists. Program participants who had received patellar bracing for the treatment of PF OA from March 2012 to July 2014 were identified as eligible for this study. These participants were assessed at the time as primarily presenting with PF OA and were treated with a Tru-Pull Lite brace (DJO Orthopaedics). Patients were prescribed a PF brace based on clinical presentation in combination with imaging. These patients were identified as those whose primary knee complaint was of anterior knee pain and symptoms, including difficulty negotiating stairs and slopes, difficulty getting up from stairs, and evidence of maltracking. These symptoms were correlated with radiographic imaging to determine if PF OA was the likely cause. Isolated PF OA was not a prerequisite.

We did not include those who had received tibiofemoral bracing or other forms of bracing treatment.

Patients were given comprehensive education by orthotists on the proper brace positioning and use, ensuring the lateral buttress of the brace was appropriately aligned with the lateral border of the patella. Initially, patients were advised to wear the brace for 1 to 2 h, and if there was no evidence of symptom flare up or skin irritation, patients were instructed to progressively increase brace use in a graded fashion. All patients were invited back for a review appointment, usually within 2 weeks, to ensure proper usage of the brace, and further bracing reviews were available if required. Follow-up reviews were performed at 12, 26 and 52 weeks, which formally examined response to treatment, and for this study the 26-week time point was used for response to intervention.

### Osteoarthritis grading and reliability

OACCP participants included in this study were assessed on the severity of their PF OA based on radiographic appearance. Non-weight bearing skyline radiographs were used. Most radiographs were obtained from the Royal North Shore radiology department, but 5 patients presented with external images. Clinician judgement was used to determine if radiographs were suitable to be included, based on patellar positioning and image quality, but no specific standardisation criteria was used. Grading was performed on the baseline images for the braced knee according to the Osteoarthritis and Cartilage grading scale for PF OA, which assesses the skyline image of the patellofemoral joint for joint space narrowing (JSN) and osteophytic changes [12]. Each patient was assessed by a single observer on two different sittings one month apart with Cohen’s kappa calculated for intra-rater reliability. Cohen’s kappa for intra-observer reliability ranged from 0.564 (moderately reliable) to 0.786 (good reliability) [13].

### Radiographic alignment measurements

**Fig. 1:**
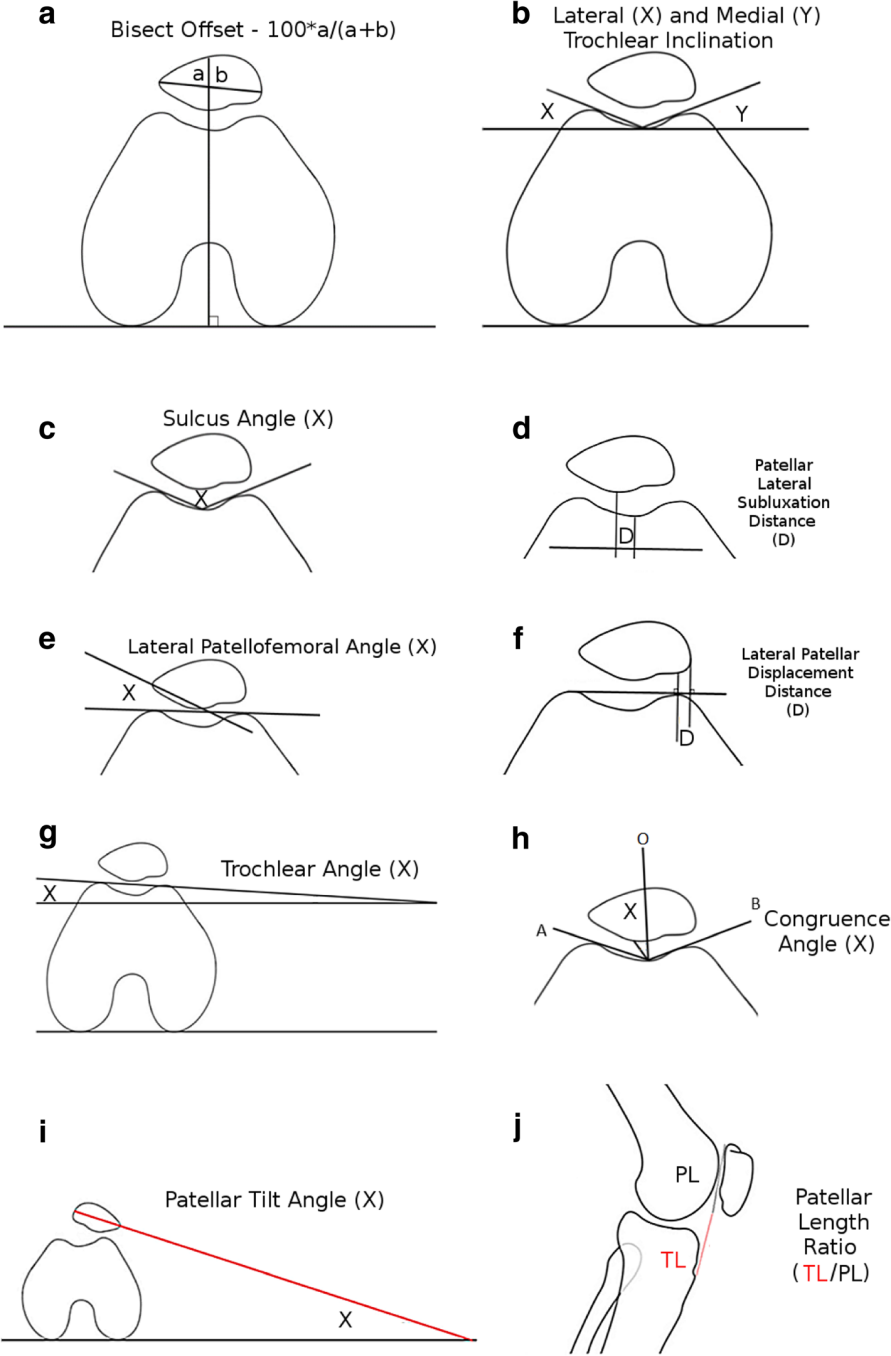
Ten measures of patellar alignment clockwise from top left: **a** bisect offset (BO); **b** trochlear inclination (TI); **c** sulcus angle (SA); **d** patellar lateral subluxation distance (PSD); **e** lateral patellofemoral angle (LPFA); **f** lateral patellar displacement distance (PSD); **g** trochlear angle (TA); **h** congruence angle (CA); **i** patellar tilt angle (PTA); **j** patellar length ratio (PLR). For congruence angle, the angle AB is bisected by O, and the congruence angle is the angle from the lowest point of the inferior margin of the patella to the line O. Note that trochlear angle is split into medial and lateral components, giving a total of eleven measurements for each patient

**Fig. 2:**
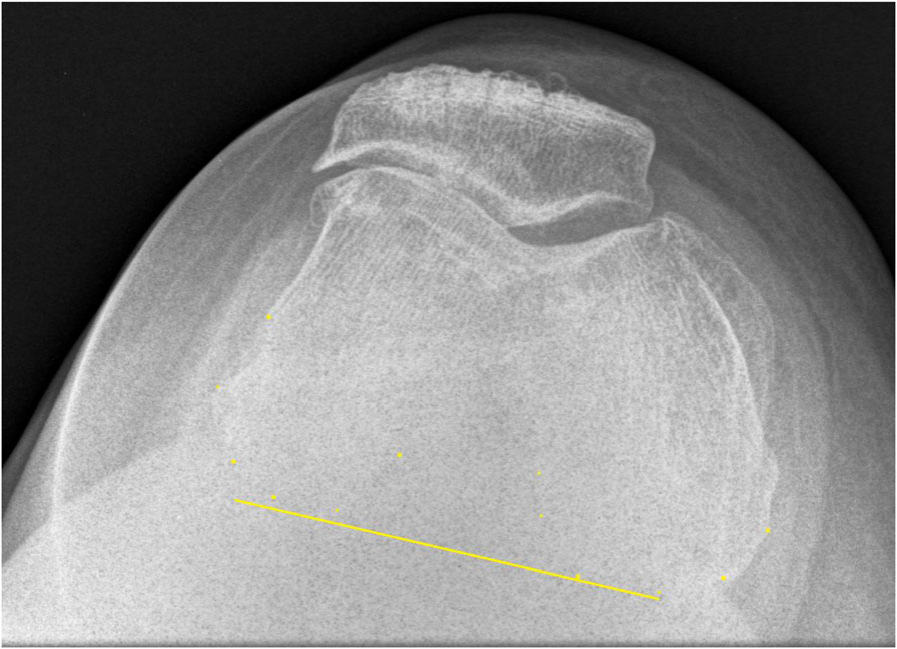
An estimate of the bisect offset. Although the posterior condylar is not visible, and estimate is made based on the visible aspects

Previous attempts at standardization of patellar alignment measures included a variety of measures to describe the position of the patella [14]. In this study, patients with lateral and patella skyline views of the symptomatic knee were examined radiographically with ten measures (Fig. 1) by a single experienced reader on two occasions. We included measures that described lateral and medial translation of the patella - bisect offset (BO) [4, 15–17], patellar lateral subluxation (PSD) distance [17], lateral patellar displacement (LPD) [17]. We also included measures for trochlear dysplasia; sulcus angle (SA) [4, 14, 15], trochlear inclination (TI) [15, 16], trochlear angle (TA) [15, 16, 18], and a measure for patellar tilt – patellar tilt angle (PTA) [4, 16, 17, 19, 20]. The modified Insall-Salvati ratio measured patellar alta and baja [15, 21, 22]. We chose the modified Insall-Salvati ratio over the traditional Insall-Salvati ratio as it has been shown to better account for differences in patellar morphology [21]. There were also measures describing the relationship between the trochlear sulcus and the patellar tilt – lateral patellofemoral angle (LPFA) [15, 17, 22], congruence angle (CA) [17, 23]. These measures have all been used in previous studies to assess patellar alignment [4, 15–23]. Many of the measures described above were initially developed for tomographic modalities, such as those that rely on the posterior condylar line (for example, bisect offset and trochlear inclination), however these modalities are costly, and, in the case of computed tomography, subjects the patient to high doses of radiation. For measures that require the posterior condylar line, an estimate based on the horizontal was made, similar to the Grelsamer et al. study, based on the appearance of the condylar alignment (Fig. 2) [19].

There have been few attempts to define a ‘normal’ range for patellar alignment measures, hence we have included the range, mean and standard deviation of all the measures we used (Table 1). Intraclass correlation for reliability (ICC) was also included, and was excellent for each measure. Some measures range from a negative value through to positive values, depending on the location of the measure – for example, a medial lying PTA was given a negative value, while the majority were lateral and were positive. The patellar lateral subluxation distance and lateral patellar displacement measures are distance measures in millimetres and thus relied on the presence of accurate scales. Of the 38 patients analysed, three presented with external images which were not to scale, and thus these measures were omitted for these patients. Tertiles for each measure was also included for statistical analysis (Table 2).

**Table 1:** Patellar alignment measures

	N	Range (S.D.)	Mean	Intraclass Coefficient (ICC)
Patellar Length Ratio (PLR)	38	1.2–2.6 (0.3)	1.7	0.997
Bisect Offset (BO)	38	50.0–100.0 (10.4)	68.4	0.948
Patellar Tilt Angle (PTA) (degrees)	38	−2.0–20.9 (5.1)	5.8	0.978
Trochlear Inclination (Lateral TI) (degrees)	38	5.2–30.6 (5.4)	21.5	0.997
Trochlear Inclination (Medial TI) (degrees)	38	10.4–36.7 (6.9)	20.9	0.997
Trochlear Angle (TA) (degrees)	38	−14.2–13.1 (6.5)	−1.6	0.937
Sulcus Angle (SA) (degrees)	38	121.3–152.5 (7.7)	137.6	0.914
Lateral Patellofemoral Angle (LFPA) (degrees)	38	.9–25.1 (6.5)	11.6	0.992
Congruence Angle (CA) (degrees)	38	−28.7–78.1 (29.9)	27.9	0.999
Patellar Lateral Subluxation Distance (PSD) (mm)	35	−3.2–11.8 (3.8)	3.2	0.998
Lateral Patellar Displacement (LPD) (mm)	35	−8.7–15.9 (5.5)	3.8	0.998

**Table 2:** Tertiles for patellar alignment measures

	Tertile 1	Tertile 2	Tertile 3
Patellar Length Ratio (PLR)	≤1.53	1.54 – 1.72	≥1.73
Bisect Offset (BO)	≤62.93	62.93 – 69.51	≥69.52
Patellar Tilt Angle (PTA) (degrees)	≤3.5	3.6 – 8.3	≥8.4
Trochlear Inclination (Lateral TI) (degrees)	≤20.1	20.2 – 24.3	≥24.4
Trochlear Inclination (Medial TI) (degrees)	≤17.2	17.3 – 23.9	≥24
Trochlear Angle (TA) (degrees)	≤ −4.8	−4.7 – 0.7	≥0.7
Sulcus Angle (SA) (degrees)	≤134.2	134.3 - 141	≥141.1
Lateral Patellofemoral Angle (LFPA) (degrees)	≤9	9.1 – 14.4	≥14.5
Congruence Angle (CA) (degrees)	≤8.6	8.7 – 47.6	≥47.7
Patellar Lateral Subluxation Distance (PSD) (mm)	≤1.4	1.4 – 5	≥5.1
Lateral Patellar Displacement (LPD) (mm)	≤1.2	1.3 – 5	≥5.1

### Symptomatic outcome measure

The Knee Injury and Osteoarthritis Outcome Scores (KOOS) is a comprehensive survey-based scoring system well validated for the assessment of OA knee symptoms [24]. This questionnaire required participants to rate their Symptoms, Stiffness, Pain, Physical Function, Recreational Activities and Quality of Life on 5-point Likert scales. The KOOS subsumes the Western Ontario and McMaster University Osteoarthritis Index (WOMAC) questions, enabling conversion to WOMAC scores. We converted each patient’s KOOS score to WOMAC score for further statistical analysis based on minimal clinically important difference (MCID). These WOMAC scores at the pre-intervention and 26-week point were retrieved from the OACCP database. The difference in WOMAC pre and post intervention was calculated, with both the raw change in score and percentage changes assigned to their respective subjects. A negative change indicated that the patient’s symptoms improved, while a positive indicated they had worsened. Each WOMAC category was included as part of analysis, consisting of Pain, Stiffness, Function and Global scores. The WOMAC Global score consists of scores from all of the domains and is scored out of 100 (zero indicating best score, 100 the worst).

In addition, patients were dichotomised into groups according to whether they were “Improved” or “Not Improved” based on the Angst et al. method of determining minimal clinically important difference [25]. The MCID is calculated depending on changes in raw score or percentage score. As this method is subject to variance depending on the initial score, we decided only patients who had met the criteria for both raw score and percentage score were marked as “Improved”. Due to the sample size, patient WOMAC scores were categorised as Improved and Not Improved, with the Not Improved group containing all patients who met the criteria for minimally important clinical worsening and those who were unchanged or improved. The Angst et al. study previously had normalised each WOMAC domain to a score of out of 10 (Table 3) [25], so we have converted each patient’s WOMAC accordingly for the purposes of MCID calculation.

**Table 3:** MCID score categories. Adapted from Angst et al. [24]

Change in raw score (% change)		
	Worsens	Improves
Pain	0.64 (14%)	−0.83 (18%)
Stiffness	0.29 (6%)	−1.01 (22%)
Function	1.03 (22%)	−0.80 (17%)
Global	0.96 (21%)	−0.82 (18%)

### Statistical analysis

**Fig. 3:**
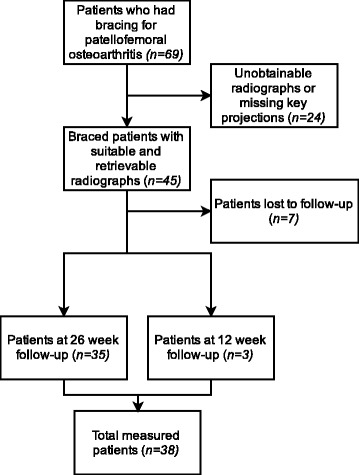
Study Sample flow chart. 69 patients were identified as eligible for participation. 24 did not have suitable radiographs and 10 were lost to 26 week follow-up. Out of these 10, three participants were present at the 12 week follow-up and they were included in statistical analysis. The final sample size was 38

There were 69 patients who met the study criteria and were suitable for inclusion. We were unable to obtain radiographs or relevant views of the patellofemoral joint for 24 patients. Of the 45 remaining, 10 patients were lost to the 26 week follow-up. Within the 10 patients lost to 26 week follow-up, three were present at the 12 week assessment, and for these patients that follow-up time point was imputed for analysis (Fig. 3). The final sample size available for statistical analysis was 38.

**Fig. 4:**
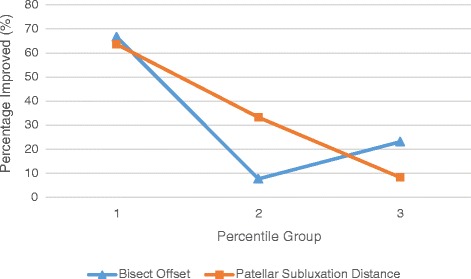
Fisher’s Exact Test for associations between percentile groups of BO and PSD and MCID categories (Global). The X axis represents each tertile group (1–3, 1 being the lowest group) for the patella parameter variable, while the Y axis represents percentage of those per tertile group that fell into the improved category

For those patients lost to follow-up, the reason for discharge was recorded. Four had joint arthroplasty, one was discharged due to program dissatisfaction, one was discharged due to other reasons, and one had not been discharged from the program and their reason was unknown. None of the three present at the 12 week assessment had been formally discharged from the program, and the reasons for non-attendance at 26 weeks was unknown.

We included sex, BMI, and the relevant knee that was measured (left or right). Spearman’s rho was used to examine correlations between radiographic measures and WOMAC scores as continuous variables. In addition to continuous variables, each measured parameter was further divided into percentiles. Fisher’s exact test was used with cross-tabulation of radiographic percentiles and MCID categories. Although dividing into radiographically normal and abnormal groups would have been preferable, the lack of standardised parameters for patellar alignment measures makes this method unfeasible. Due to the low sample size, tertiles were chosen instead of quartiles to improve statistical power. We also performed linear regression with the dependent variable as change in WOMAC scores and the independent variable as patellar alignment parameters. Simple linear regression was used instead of multivariate regression due to the low sample size.

As the OACCP assessment did not assign separate KOOS questionnaires to individual knees, patients with bilateral bracing were indexed based on initial assessment notes that marked the most symptomatic knee. In three patients, the most symptomatic knee was not listed, so the knee with the most severe osteoarthritis grading was chosen as the indexed knee. Both MCID categories and the original raw change in WOMAC scores were used as dependent variables, with independent variables being the patellar alignment measures, as well as tertiles for each measure.

## Results

For the 38 patients analysed, their age ranged from 51 to 89, with a mean of 67.2 years. There was a female predominance of 76.3% and BMI ranged from 20.8 to 45 kg/m2, with an average value of 29.2 kg/m2. The left side was the more commonly indexed knee, at 55.3%. Global WOMAC scores ranged from −25 to 41.67, with a mean change of −3.97. Despite the mean value of change in WOMAC scores indicating reduction in symptom severity, most patients did not meet the threshold for MCID, with 31.6, 44.7 and 31.6% of patients falling into the “Improved” group for Global, Pain and Function scores respectively (Table 4). Radiographic grading for the indexed knee demonstrated that lateral patellofemoral compartment osteoarthritis was more common than medial, with 76.3% of patients having grade 0 joint space narrowing (JSN) in the medial patellofemoral compartment (indicating minimal appreciable JSN) [11], and no patients were observed to have grade 3 medial compartment JSN. Lateral compartment JSN scores were more evenly distributed, with only 26.3% having grade 0 JSN, and 63.1% as having either grade 2 or 3 lateral JSN. Lateral osteophytes were also more likely to be severe than medial, with 50% of participants having grade 2 or 3 osteophytes in the lateral compartment, and 36.9% of participants having grade 2 or 3 osteophytes in the medial compartment.

**Table 4:** Demographics, mean WOMAC scores and grading frequencies (*n =* 38)

	Value	Range (S.D.)
Female (%)	76.3	
Age (mean years)	67.2	51 to 89 (7.96)
BMI (mean kg/m^2^)	29.2	20.8 to 45 (5.13)
Change in WOMAC Global (mean)	−3.97	−25 to 41.67 (13.07)
Change in WOMAC Pain (mean)	−1.53	−14 to 8 (3.49)
Change in WOMAC Function (mean)	−1.87	−18 to 32 (9.49)
MCID Improved (Global) (%)	31.6	
MCID Improved (Pain) (%)	44.7	
MCID (Improved (Function) (%)	31.6	
Affected knee (%)		
Left	55.3	
Right	44.7	
Medial Joint Space Narrowing category (%)		
0	76.3	
1	15.8	
2	7.9	
3	0	
Lateral Joint Space Narrowing category (%)		
0	26.3	
1	10.5	
2	34.2	
3	28.9	
Medial Osteophytes Category (%)		
0	31.6	
1	31.6	
2	23.7	
3	13.2	
Lateral Osteophytes Category (%)		
0	15.8	
1	34.2	
2	23.7	
3	26.3	

### Correlations between alignment parameters and WOMAC groups

Initial scatter plots and two-tailed Spearman’s rho did not reveal any correlation between patellar alignment measures and change in WOMAC scores. There was a non-significant trend shown (*p =* 0.058) between bisect offset and change in WOMAC global, with a correlation coefficient of 0.31, indicating a trend for higher change in WOMAC scores with increasing bisect offset. Spearman’s rho correlation coefficients ranged from −0.23 to 0.31 for each measure when correlated against WOMAC global (Table 5).

**Table 5:** Spearman’s rho test for patellar alignment measures and change in WOMAC global

			Change In WOMAC Global
Spearman’s rho	Patellar Length Ratio	Correlation Coefficient	.184
		Sig. (2-tailed)	.268
		N	38
	Bisect Offset	Correlation Coefficient	.311
		Sig. (2-tailed)	.058
		N	38
	Patellar Tilt Angle	Correlation Coefficient	.089
		Sig. (2-tailed)	.594
		N	38
	Trochlear Inclination (medial)	Correlation Coefficient	–.060
		Sig. (2-tailed)	.720
		N	38
	Trochlear Inclination (lateral)	Correlation Coefficient	–.160
		Sig. (2-tailed)	.337
		N	38
	Trochlear Angle	Correlation Coefficient	—.215
		Sig. (2-tailed)	.194
		N	38
	Sulcus Angle	Correlation Coefficient	.164
		Sig. (2-tailed)	.325
		N	38
	Lateral Patellofemoral Angle	Correlation Coefficient	–.236
		Sig. (2-tailed)	.153
		N	38
	Congruence Angle	Correlation Coefficient	.228
		Sig. (2-tailed)	.168
		N	38
	Patellar Lateral Subluxation Distance	Correlation Coefficient	.276
		Sig. (2-tailed)	.109
		N	35
	Lateral Patellar Displacement Distance	Correlation Coefficient	–.024
		Sig. (2-tailed)	.890
		N	35

### Correlations between alignment percentile groups and MCID groups

We used contingency tables to analyse the association between percentile scores and MCID categories. The improved groups often did not meet the expected frequency of five for the chi-square test per percentile division, therefore Fisher’s exact test was used (Table 6). There was a statistically significant association between BO (*p <* 0.01) and Global MCID and Function MCID, suggesting that higher BO groups were more likely to be associated with the “Not Improved” group. PSD was associated with Global and Function MCID (*p <* 0.05), again suggesting higher percentile groups were associated with the “Not Improved” group (Fig. 4).

**Table 6:** Fisher’s Exact Test for associations between percentile groups of alignment measures and MCID categories (Global)

	Percentile Groups Global Improved (%)			
Variable	Lowest tertile	Middle tertile	Highest tertile	Fisher's Exact Test
Patellar Length Ratio (PLR)	3(25)	5(38.5)	4(30.8)	*p =* 0.907
*Bisect Offset (BO)*	*8(66.7)*	*1(7.7)*	*3(23.1)*	*p =* *0.007*
Patellar Tilt Angle (PTA)	5(41.7)	5(38.5)	2(15.4)	*p =* 0.323
Trochlear Inclination (TI) (Lateral)	4(33.3)	3(23.1)	5(38.5)	*p =* 0.757
Trochlear Inclination (TI) (Medial)	2(16.7)	6(46.2)	4(30.8)	*p =* 0.383
Trochlear Angle (TA)	3(25)	5(38.5)	4(30.8)	*p =* 0.907
Sulcus Angle (SA)	4(33.3)	4(30.8)	4(30.8)	*p =* 1
Lateral Patellofemoral Angle (LPFA)	2(16.7)	6(46.2)	4(30.8)	*p =* 0.383
Congruence Angle (CA)	5(41.7)	5(38.5)	2(15.4)	*p =* 0.323
*Patellar Lateral Subluxation Distance (PSD)*	*7(63.6)*	*4(33.3)*	*1(8.3)*	***p =*** *0.023*
Lateral Patellar Displacement (LPD)	3(27.3)	5(41.7)	4(33.3)	*p =* 0.903

BO: p = 0.007

PSD: p = 0.023

For each tertile group, the columns indicate the number (and percent) of that tertile group that fell into the MCID improved category

### Linear regression

In single independent variable linear regression modelling, there was a statistically significant association between change in WOMAC Function and trochlear angle, with a smaller angle being predictive of lower scores, indicating improvement (R [2] = 0.104, F(1,36) = 4.168, *p =* 0.049). However, due to absence of similar results for trochlear angle on Spearman’s correlation, this was most likely a false positive. No other significant models were demonstrated.

### Secondary analysis

We performed secondary analyses with the 5 patients who were discharged due to surgery or non-satisfaction as part of the “not improved” group. Again, higher percentile groups were more likely to be “not improved.” Fisher’s exact test for WOMAC global MCID scores were *p =* 0.032 and *p =* 0.008 for BO and PSD respectively. This appears to support our findings in our primary analysis.

## Discussion

Patellofemoral osteoarthritis is a common condition however there is little consensus on non-surgical approaches to management [6, 7, 26, 27]. Contrary to our hypotheses, our results suggest an inverse relationship between patients with bisect offset and patellar lateral subluxation distance and bracing as a treatment for symptoms of patellofemoral osteoarthritis, with those who fell in the highest tertile tending to respond the most poorly.

Previous studies on the efficacy of bracing as a treatment for PF OA have reported mixed results [6–8, 11], however bracing is still a common conservative option. Bracing is an inexpensive, non-invasive intervention that is widely available, and thus it is in public interest to define its therapeutic reliability and limitations. As alignment is an important factor in PF OA progression and symptoms, a treatment that addresses these factors would be logical and of theoretical benefit [3, 4]. A systematic review of patellofemoral bracing for pain did not find any benefit in knee braces for treating anterior knee pain, however most studies were short term (ranging from immediate same-day time frame to 3 to 12 weeks) and none focussed on pain from OA [6]. Hunter et al. examined bracing in a randomised-controlled trial that compared a brace with a realigning strap with a non-realigning brace; the results failed to demonstrate a difference between the two groups [7]. Callaghan et al. [11] in a more recent randomised trial did show a statistically significant improvement in symptoms for patients with PFOA who received bracing compared to those that received no brace over a 6 week period [27]. Although overall our bracing cohort demonstrated small improvements across all measured domains, only 44.7% of patients met the mean clinically important difference threshold for improvement in pain symptoms and 31.6% for global symptoms.

Although we hypothesised that more severe malalignment was likely to affect the efficacy of bracing, the direction of effect was unknown. Current models of patella kinematics suggest that bracing compresses the patella to be seated more firmly in the trochlear sulcus, correcting patella maltracking and leading to more evenly distributed contact area with reduced joint stress [9, 11]. We had hypothesised that extreme patellar malalignment would benefit more from bracing than those with less, but found that those in higher percentiles of malalignment were less likely to fall into the “Improved” group.

The reason for this may be manifold. While patella malalignment has been positively linked to progression of PFOA [4, 28, 29], malalignment itself may not be sufficient enough to cause pain [5, 17, 30], and correction of malalignment will not necessarily reduce symptoms. [30, 31] The current understanding of the aetiology of pain deriving from patellar malalignment is incomplete [30, 31]. One accepted hypothesis theorises that increased joint stress from lateral patella tilt and translation may be responsible for pain [31, 32]. Previous research on patellar taping for chronic pain has shown positive results [6, 8], and the correction of this lateral translation and tilt has been one proposed mechanism for its therapeutic benefit. However, Crossley et al. noted that their patellar taping study also included a mechanism to unload the infrapatellar fat pad, one of the most sensitive pain-producing areas in the knee, which may account for the higher beneficial effect in that study [8].

None of the patients in our study had a bisect offset under 50 (Table 1), and this suggests that patellar translation in our study was purely lateral, not medial. Bracing also corrects for this malalignment, however the kinematic effects of bracing appears to be different to that of taping [8, 10, 33–36]. Previous research on the effects of bracing on patellofemoral joint alignment found that correction of bisect offset ranged from 1 – 6% [10, 33–36], while the effect of taping was minimal at under 1% [8]. In addition, previous research has shown that bracing increased joint surface contact area by up to 21 – 41%, depending on stance and position, and reduced contact stress by 17 – 27% [34]. However, patellar tilt does not appear to be significantly corrected in bracing compared to taping; Muhle et al. found bracing had little effect on lateral patellar tilt angle [34], compared to Crossley et al. who found a significant 3.57° increase in LPTA [8].

The disparate results may suggest that patellar tilt is more closely linked to symptoms rather than medial or lateral translation. We speculate that bisect offset and PSD (which are measures of medial and lateral translation) are not corrected enough for patients with more severe malalignment, and thus only the least affected patients benefit from the modest alignment correcting effects of bracing. This explains why in our study, patients whose BO and PSD fell into more extreme groups fared more poorly than those with more moderate medial and lateral translation of the patella. Other parameters did not appear to modify the effectiveness of bracing, although this may be due to the limitations of our study Future studies should continue to examine the relationship between bracing and other patellar alignment measures, including the measures we included in our study.

Our study examined the relationship between numerous patella alignment variables, with inclusions for multiple descriptors of patella morphology. Plain radiographs were used in our study, which allows for easy repeatability due to the low cost and wide availability of this modality, as well as the low ionising radiation dose compared to CT imaging.

There are several limitations to our study to consider. For data collection, assumptions for three patients were made on the symptomatic knee based on radiographic appearance as the most symptomatic knee was not listed, which may not reflect the actual most affected joint. Three participants were also omitted for the measures of PSD and LPD due to lack of scale, which reduces the sample size of those measures. Three participant outcomes were analysed based on the 12 week follow-up rather than the 26 week as they did not return. The outcome measures may not be accurate for these patients as the bracing intervention may be affected by length of treatment time. In addition, although our patients were reviewed for compliance and adherence by orthotists and physiotherapists, there were no specific metrics taken to ensure this. Another potential confounder is that each patient was also prescribed dietary, weight management and exercise advice, which may limit the interpretation of our results.

The small sample size reduces the power of this study, and multivariate regression is limited by this as well. In addition, some measures relied on the posterior condylar line, which cannot be determined on a plain skyline radiograph. We have made estimates based on the image, and other measures do not rely on the posterior condylar line, but further studies should also consider 3D-tomographic modalities such as CT or MRI instead of plain radiographs as they may allow for more accurate measuring of patellar alignment. Future studies examining the relationship between patellar malalignment and bracing should increase the sample size, which may reveal correlations that this study lacked statistical power to interpret. Randomised controlled studies would be the preferred study design, though study cost may be a limiting factor. Increasing sample size would also allow for multivariate regression, to examine whether certain combinations of patellar alignment parameters affect the efficacy of bracing more than single parameters. Future studies should also examine the degree of patellar alignment correction with patellofemoral bracing in OA to examine if those with greater degrees of patellar malalignment achieve greater levels of correction to alleviate symptoms.

## Conclusion

These results suggest patients with greater patellofemoral lateral translation respond more poorly to bracing as a form of treatment for patellofemoral osteoarthritis, compared to patients with less severe alignment. This study is the first to examine the relationship between patellar alignment and bracing as a treatment for OA. Further studies are required to examine this relationship.

## Abbreviations

BO: Bisect offset
CA: Congruence angle
JSN: Joint space narrowing
KOOS: Knee Injury and osteoarthritis outcome scores
LPD: Lateral patellar displacement
LPFA: Lateral patellofemoral angle
MCID: Mean clinically important difference
OA: Osteoarthritis
OACCP: Osteoarthritis chronic care program
PF: Patellofemoral
PFOA: Patellofemoral osteoarthritis
PLR: Patellar length ratio
PSD: Patellar lateral subluxation distance
PTA: Patellar tilt angle
SA: Sulcus angle
TA: Trochlear angle
TI: Trochlear inclination
WOMAC: McMaster University Osteoarthritis index
